# What About the Children? Co-Occurrence of Child Maltreatment and Parental Separation

**DOI:** 10.1177/10775595221130074

**Published:** 2022-09-24

**Authors:** Sheila R. van Berkel, Mariëlle J. L. Prevoo, Mariëlle Linting, Fieke Pannebakker, Lenneke R. A. Alink

**Affiliations:** 1 Institute of Education and Child Studies, 100575 Leiden University, Leiden, The Netherlands; 2 Research Support and Development, University Library, 5211 Maastricht University, The Netherlands; 3 Child Health, 2859 TNO, The Netherlands

**Keywords:** parental separation, high conflict divorce, child maltreatment, interparental violence

## Abstract

The aim of this study was to investigate (a) the extent to which child maltreatment co-occurs with parental separation and (b) associations between different types of child maltreatment and various types of separation-associated interparental conflict. Professionals working with children (*N* = 785) reported each case of suspected child maltreatment they observed during a 3-month period and indicated whether parental divorce or separation was about to take place or had taken place. This resulted in 530 reported cases that matched the definitions of child maltreatment for which information on parental relationship status was available. Most of the maltreated children (60%) also experienced (impending) parental separation. In 69% of these cases child maltreatment was associated with parental separation. Particularly, cases of emotional neglect, and emotional abuse co-occurred with parental separation. In addition, four clusters of separation-associated interparental conflict were distinguished— *No observed conflict*, *Non-physical conflict*, *Verbal and physical conflict*, and *Multiple conflict—*which were associated with child and family characteristics and specific types of child maltreatment. The results of this study suggest that child maltreatment often co-occurs with parental separation, especially when there is a considerable amount of interparental conflict.

Child maltreatment poses a considerable risk for detrimental outcomes in multiple developmental domains (Cicchetti & Toth, 2005). Among the many family, parent and child characteristics associated with child maltreatment, aspects related to parental separation or divorce seem to play an important role, such as single parenthood, living with a stepparent, and interparental violence (Assink et al., 2019; Mulder, et al., 2018; Stith et al., 2009; Van Berkel et al., 2020). It is surprising that the relation between child maltreatment and parental separation has received little attention in the literature, because single parenthood and living with a stepparent are both clearly connected to parental separation as well as interparental violence (Holtzworth-Munroe, 2011; Van Dijk et al., 2020). This association is particularly relevant considering evidence that parental separation can also negatively affect child development (Auersperg et al., 2019). The current study aims to provide insight into the extent to which parental separation (including divorce) co-occurs with child maltreatment in general and its specific types and into possible risk factors for separation-associated child maltreatment. Knowledge on the association between parental separation and child maltreatment can be beneficial for tailoring preventive interventions at divorced or separated families.

Parental separation (defined as the termination of the relationship between a child’s parents regardless of whether parents were married) is for many children a traumatic experience in itself and has been associated with numerous negative long-term outcomes on academic achievement, mental health, delinquency, self-esteem, and interpersonal relations (Lansford, 2009; Sands et al., 2017; Van Dijk et al., 2020). Detailed international data from official sources on the prevalence of separation of couples with children are lacking. However, a cross-national comparative study on family dynamics based on National survey data of the US, Russia and 17 European countries indicates that in these countries 10–44% of the couples with children had separated before one of their children reached the age of 15 years (Andersson et al., 2017). In addition to the negative outcomes on child development, parental separation may enhance the risk for child maltreatment (Afifi et al., 2009; Baker & Ben-Ami, 2011; Dong et al., 2004), even though maltreatment does not occur in the majority of separated families. There are several explanations for the association between parental separation and maltreatment, which will be discussed in more detail: factors related to parental separation may increase the risk of parenting problems and maltreatment, high conflict parental separation may be maltreatment in itself, the same underlying risk factors may explain both parental separation and maltreatment, and child maltreatment may be a risk factor for parental separation.

Parental separation can be considered a turbulent transitional period during which several disruptions take place in family life (Van Dijk et al., 2020). In addition to changes in living situation and amount of contact with (one or both of the) parents, these often include increased interparental conflict, disruption of the parent-child relationship and parenting quality, and declined economic resources (Amato, 2010; Lansford, 2009). The extent of these disruptions has been related to variations in child outcomes following parental separation. In addition to the direct threat these disruptions pose on the child’s development, they also have been related to an increased risk for child maltreatment (Assink et al., 2019; Mulder, et al., 2018; Stith et al., 2009; Van Berkel, et al., 2020). Separation often involves a decline in economic resources and social support, for the resident parent (often the mother) single parenthood may be a challenge, while for the non-resident parent (often the father) the lack of daily contact with the children may result in feelings of loss, helplessness, inadequacy, and feelings of incompetence (Amato, 2000; Coley, 2006; Kruk, 2010). Several studies found that parents report more depression symptoms, and experience more social isolation and health problems after separation (e.g., Bierman et al., 2006; Wood et al., 2007). These stressors may consume the parents energy, resulting in less optimal parenting (Altenhofen et al., 2010; Elam et al., 2019). This is in line with the *Family Stress Model* (Masarik & Conger, 2017) that predicts that the stress, irritability, anger, and frustration resulting from separation-related circumstances can lead to more hostile and harsh parenting strategies which are risk factors for child maltreatment. Based on this model, the high-conflict nature of part of parental separations may also influence parenting quality and increase the risk on child maltreatment (Polak & Saini, 2019; Van Dijk et al., 2020). This idea is confirmed by several studies showing that the link between interparental conflict and negative child outcomes (such as psychopathology, negative self-esteem, internalizing and externalizing problems) is, at least partially, mediated by parenting quality (Harold & Sellers, 2018; Van Dijk et al., 2020) and studies showing that interparental violence and child maltreatment often co-occur (Assink et al., 2019; Guedes et al., 2016; Schellingerhout & Ramakers, 2017; Van Berkel et al., 2020).

Increases in parental conflict associated with the challenges and disturbances of the transition that parental separation holds often start before the actual separation takes place and continue on average 2–3 years post-separation (Kelly, 2007; King & Heard, 1999). In about 10–15% of the parental separations, however, these conflicts continue despite the passage of time and can go on for even longer periods of time (Amato, 2001). Parental separations that are characterized by continuing and apparently unresolvable conflicts between the ex-spouses, have been defined as high-conflict or complex separations (Amato, 2001; Grych, 2005; Smyth & Moloney, 2009; Stewart, 2001). The international literature acknowledges that high-conflict separations are related to risk factors for child maltreatment such as parenting problems and lack of supervision, as well as directly to child maltreatment, such as child emotional neglect (Joyce, 2016; Sturge-Apple et al., 2006; Van Dijk et al., 2020). The Dutch guidelines for youth care and youth protection (2020) go even a bit further by stating that parents involved in a complex separation ‘*lose sight of the interests and welfare of their children due to the persistent and severe conflicts*’. This definition of complex separations thus includes parental failure to meet children’s emotional needs and to provide adequate affection, which is considered emotional neglect (Dullaert, 2014; Sedlak, et al., 2010; Stoltenborgh et al., 2013). Separation-related conflict between parents may include legal disputes, ongoing disagreement over day-to-day parenting practices, custody and visitation arrangements, and denigration of or hostility towards the other parent in front of the children (Johnston & Roseby, 1997; Smyth & Moloney, 2009). Given this wide array of conflict types now all defined by *high conflict* separation, it has been suggested that empirical research is needed to identify various types of high-conflict separations (Birnbaum & Bala, 2010; Cashmore & Parkinson, 2011). Moreover, a better understanding of differences between families involved in different types of separation-related conflicts may provide a better insight in the different risk factors that may contribute to separation-related conflicts (Polak & Saini, 2019), as well as in variations in child outcomes and the co-occurrence with child maltreatment (Grych, 2005; Polak & Saini, 2019). Therefore, the current study aimed to identify separation-related conflict clusters and examined how these clusters were associated with different forms of child maltreatment.

Furthermore, high levels of separation-related interparental conflict often include verbal or physical interparental violence (Johnston & Roseby, 1997). A study with children in contested custody cases indicated that 41% of these children had witnessed interparental violence (Hirst, 2002). Moreover, a history of interparental violence has been related to an increased risk of a high-conflict separation (Polak & Saini, 2019). Witnessing such interparental conflicts and/or violence is in itself child maltreatment as this is considered a form of emotional neglect (Dullaert, 2014; Sedlak, et al., 2010; Stoltenborgh et al., 2013).

It is also possible that the same risk factor underlies both parental separation as well as child maltreatment. Several factors have been shown to increase the risk of both, such as parental psychopathology and substance abuse, interparental violence, child problem behavior, and low parental education (Amato, 2012; Mulder, et al., 2018; Stith et al., 2009; Wade & Pevalin, 2004; Wymbs et al., 2008). Furthermore, child maltreatment may be a risk factor for parental separation. Allegations of child maltreatment are regularly made by parents in court during child custody cases and there is some evidence that at least half of these allegations are legitimate (Johnston et al., 2005; Trocmé & Bala, 2005).

Although several studies have investigated family factors that may be related to child maltreatment, research on parental separation in maltreating families is scarce and it is still unknown how frequently parental separation has taken place or is about to take place in families in which child maltreatment occurs. To provide insight in the extent to which maltreating families experience parental separation and better understand the co-occurrence of parental separation and child maltreatment, the current study investigates (1) the prevalence of parental separation in a sample of children reported by informants for unsafe home situations that matched the definitions of child maltreatment. Moreover, we investigated (2) whether the reported child maltreatment was associated with parental separation and (3) compared the percentages of separation-associated child maltreatment for the different types of child maltreatment. We expected that particularly emotional neglect and emotional maltreatment would be associated with parental separation. In addition, (4) specific known child- and family-level risk factors for child maltreatment—such as parental low educational levels, parental psychopathology and drug use, parental unemployment, immigrant background, stepfamilies, single-parent families, a large number of children in the family, and young child age (i.e. younger than 4 years of age; Assink et al., 2019; Mulder et al., 2018; Sedlak et al., 2010; Stith et al., 2009; Van Berkel et al., 2020)—were investigated as possible risk factors for separation-associated child maltreatment specifically. We expected to find that these risk factors would also increase the risk on separation-associated child maltreatment. Finally, (5) associations between variation in the type of separation-associated interparental conflict (e.g., physical conflict, loyalty conflicts, legal conflicts) and the occurrence of different types of child maltreatment were investigated, as was the occurrence of the possible risk factors described above.

## Method

### Participants

Organizations and participating professionals within these organizations—sentinels—were randomly selected within several occupational branches: child care and kindergarten, primary and secondary schools, well-baby clinics, general practitioners, child protection professionals of hospitals, shelters for battered women and Child Protection Boards^1^ (for a more detailed description of the recruitment see Van Berkel et al., 2020). To ensure a geographically representative sample, provinces in the Netherlands were arranged in five zones with approximately equal numbers of children living in each zone. The number of sentinels to be included within each occupational group was determined in proportion to the number of children in that zone. Nonresponse was handled by randomly selecting new organizations and professionals in a given zone to prevent selection bias. In total, 785 professionals from 289 organizations participated in the study (Table 1).

**Table 1.** Total Numbers of Participating Organizations and Professionals, Sample of Observed Children, and Total Dutch Population Per Occupational Branch.

	Total number of organizations	Total number of professionals	Sample of observed children^b^	Total Dutch population
Home-based and center-based child care	80	133	2,708	531,450
Kindergartens	30	41	1,266	47,000
Primary schools	45	272	6623	1,427,506
Secondary schools	21	65	1,825	995,725
Well-baby clinics	18	113	24,477	783,154
General practitioners	33	37	21,974	3,404,098
Child protection professionals in hospitals^a^	36	36	1,753,425	3,404,098
Shelters for battered women	16	39	1,840,053	3,404,098
Child protection boards	10	49	130,331	3,404,098
Total	289	785		3,404,098

^a^Specialized in the evaluation of and response to child maltreatment.

^b^The samples of observed children cannot be summed to a total, since children can be observed by more than one occupational branch.

### Procedure

Sentinels filled out a standardized online registration form, based on the form used in the NIS studies (National Incidence Study of Child Abuse and Neglect; Sedlak et al., 2010) and previous Netherlands Prevalence Study of Maltreatment of Youth (NPM) studies (NPM-2005: E.M. Euser et al., 2010 and NPM-2010: S. Euser et al., 2013) for each child of their professional population for whom they suspected child maltreatment during a 3-month period, from mid-September to mid-December 2017. The registration form consisted of questions about the child, the home situation, the caregivers of the child, the suspected maltreatment, suspected perpetrators, and the context of domestic violence and parental separation or divorce. Since it was plausible that sentinels did not know all details of the families they reported on, all questions in the registration form had the response option ‘unknown’. Due to very recent reorganizations at the Child Protection Boards, participating professionals from these organizations considered filling out the registration form as too much of a burden. Therefore, the procedure was adapted for this occupational branch such that research assistants filled out the registration forms based on the information in the client files of the selected sentinels of the Child Protection Boards (CPBs). This method was chosen since professionals of the CPBs indicated that they would have used the information in the files themselves when filling out the registration forms, since these client files comprise the description of the child’s home situation and situations that may jeopardize the child’s safety. We compared the amount of missing data to investigate possible differences between the forms of the CPBs (filled out by research assistants) and other occupational branches (filled out by sentinels). Forms completed based on files of CPBs contained more background information on parents’ employment, educational level, immigrant status, and parental separation (*p*s < .004), which can probably be explained by the fact that CPBs register more information about the family compared to the other occupational branches and not necessarily by differences in the method of data collection.

Sentinels reported child maltreatment concerning 735 children (Van Berkel et al., 2020). Thirty-two cases were removed because the victim was in utero or 18 years of age or older, the maltreatment did not take place during the designated period, or because the child did not belong to the sentinel’s population (e.g., an older sibling of a child from a sentinel of a well-baby clinic). Additionally, 40 were removed because the case descriptions were not considered maltreatment (see the description of coding of maltreatment below). Examination of the data for duplications revealed two cases that were reported by two different sentinels. The two registration forms of these children were integrated to one form. This resulted in a final sample of 663 reported cases that matched the definitions of child maltreatment of children living in 472 families. In the Netherlands, it is not mandatory to report suspicions of child maltreatment. However, in most of these cases (75.6%) the sentinels indicated that they had also reported this case to Safe at Home organizations (the National contact point for domestic violence and child maltreatment). In 68.3% of the cases that were not presented to Safe at Home, the family already received professional care or sentinels arranged professional care without the help of Safe at Home.

### Coding of Maltreatment

The detailed descriptions of situations reflecting potential child maltreatment and unsafe home situations provided by sentinels were independently coded by three trained coders to (a) decide whether the cases were considered child maltreatment following the definitions used in the previous NPM-studies (E.M. Euser et al., 2010; S. Euser et al., 2013) and the NIS-4 (Sedlak et al., 2010) and (b) to classify the description into one or more of six maltreatment types: (1) sexual abuse, (2) physical abuse, (3) emotional abuse, (4) physical neglect, (5) emotional/educational neglect, (5a) witnessing interparental violence (as a specific subtype of emotional neglect) and (7) unspecified abuse or neglect. This last category was coded when there was clearly maltreatment but the information to specify any of the other subtypes was insufficient. To guarantee consistency with the coding of the NPM-2010, intercoder reliability between a coder involved in coding for the NPM-2010 and the expert coder of the NPM-2017 was first assessed. Reliability estimates (kappa) ranged from .75 to .93 for the different types of maltreatment. Next, a reliability set of 93 forms (from the NPM-2010 and the NPM-2017) was coded by all three coders, and intercoder reliability ranged from .65–.95. In case of doubt, the case was discussed with the other coders to reach consensus.

### Coding of Parental Separation

The cases of maltreatment of which sentinels reported that parents were divorced or separated without having been married or were about to get a divorce/separation (*n* = 314) were coded by trained coders. Based on the detailed description of the maltreatment and family circumstances provided be the sentinels, coders determined (a) whether parental relationship status was (proximally or distally) associated with the reported maltreatment and (b) what type of conflicts between parents were reported by sentinels. Parental separation was considered proximally associated with child maltreatment when the reported maltreatment was linked to the separation, for example emotional neglect originated from loyalty conflicts imposed by parents after or in the process of getting a separation (e.g., involving children in mutual parental accusations). Parental separation was coded as distally associated when circumstantial changes resulting from the parental separation were associated with the maltreatment, for example if emotional neglect originated from the problems of a single parent to manage the family after a separation. In addition, described conflicts were classified into eight categories: (1) physical conflicts (all forms of physical violence between parents, for example hitting or throwing objects towards the other parent), (2) verbal conflicts (e.g., threatening, shouting), (3) legal conflicts (e.g. conflicts concerning custody arrangements, alimony), (4) loyalty conflicts (e.g. descriptions of one parent denigrating the other parent towards the children, or preventing children to have contact with the other parent), (5) parenting conflicts (e.g. disagreements on rules and expectations for their child), (6) other conflicts (conflict was present but information to classify it in the other categories was insufficient), and (8) unknown (which was coded if no information was provided on conflicts between parents). Since these categories were not mutually exclusive, conflicts could be coded in more than one category (e.g. conflicts between parents could include verbal and physical violence and consider parenting disagreements). Two trained coders independently coded the reported cases in which a separation between parents was about to take or had taken place. A reliability set of 30 forms was coded by the two coders, and intercoder reliability ranged from .71–1.00. In case of doubt, the case was discussed to reach consensus.

**Types of conflictual parental separation.** To determine if parental separations could be classified into different conflict profiles, a two-step cluster analysis was conducted on five of the coded conflict categories using SPSS (i.e. physical conflicts, verbal conflicts, legal conflicts, loyalty conflicts, parenting conflicts). As distance measure, the log-likelihood criterion was used and Akaike’s Information Criterion (AIC) and the silhouette coefficient were applied to compare cluster solutions, with silhouette coefficients above 0.50 considered good solution quality (Sarstedt & Mooi, 2014).The categories *other conflicts* and *no conflicts* were coded only 2 or 3 times and were therefore not included in the cluster analysis. Four distinct parental conflict clusters were identified: (1) the *No observed conflict* group (*n* = 99, 31%) with families for whom none of the conflict categories were reported, (2) the *Non-physical conflict* group (*n* = 81, 26%) with families for whom all conflict categories except the physical conflicts were reported, (3) the *Verbal and physical conflict* group (*n* = 78, 25%) with families for whom only verbal and physical conflicts were reported, and (4) the *Multiple conflict* group (*n* = 56, 18%) with families for whom all five conflict categories were reported. Cluster quality was good with a silhouette coefficient of 0.70.

### Data Analyses

Frequencies of parental separation in child maltreatment cases were computed for each type of maltreatment and for the different clusters of parental conflict. Differences in the occurrence of the specific types of child maltreatment between parental conflict clusters were examined with chi-square tests and Bonferroni-corrected post-hoc pairwise comparison. To test child and family characteristics as potential risk factors for parental separation-associated child maltreatment, differences in the occurrence of family and child characteristics between cases of separation-associated child maltreatment and child maltreatment not associated with separation were examined with chi-square tests and Bonferroni-corrected post-hoc pairwise comparison.

Several risk factor variables contained considerable amount of missing data since specific information on the family was not known by the participating professionals. Especially demographic information of the parents had a large number of missing values: 81% of parental education, 62% of parental psychopathology (including drug use), 58% of parental unemployment, and 49% of immigration status had a missing value. Other risk factors showed fewer missing values: family composition (i.e., single parent family or step-family) contained 6% missing values, number of children in the household 10% missing values, and child age 7% missing values. Missing values on parental separation, immigration status, parental education, unemployment, and number of children in the family were associated with the occupational branch of the participating professional (*p*s < .001). This can be explained by differences between the branches in what kind of family information is regularly asked and registered. For example, information on parental unemployment and parental education was more often missing in forms filled out based on CPB-case reports than forms filled out by professionals of well-baby clinics and secondary-school teachers respectively, while information on immigration status was more often missing in forms of child protection professionals of hospitals compared to forms filled out based on CPB-case reports, or by primary-school teachers and professionals of shelters for battered women. Pairwise deletion (available-case analysis) of missing data was applied and risk factors with more than 50% missing values were excluded from the analyses. This resulted in the inclusion of the following risk factors: (1) immigrant status (first-generation—being born outside the Netherlands—or second-generation—having at least one first-generation immigrant parent; NPM-2017; Van Berkel et al., 2020), (2) large family size (defined as families with four or more children), (3) single parenthood (4) stepfamilies, and (5) young age of the child (3 years or younger). Similarly, possible risk factors for specific parental conflict clusters within separation-associated child maltreatment were investigated.

## Results

**Figure 1.**
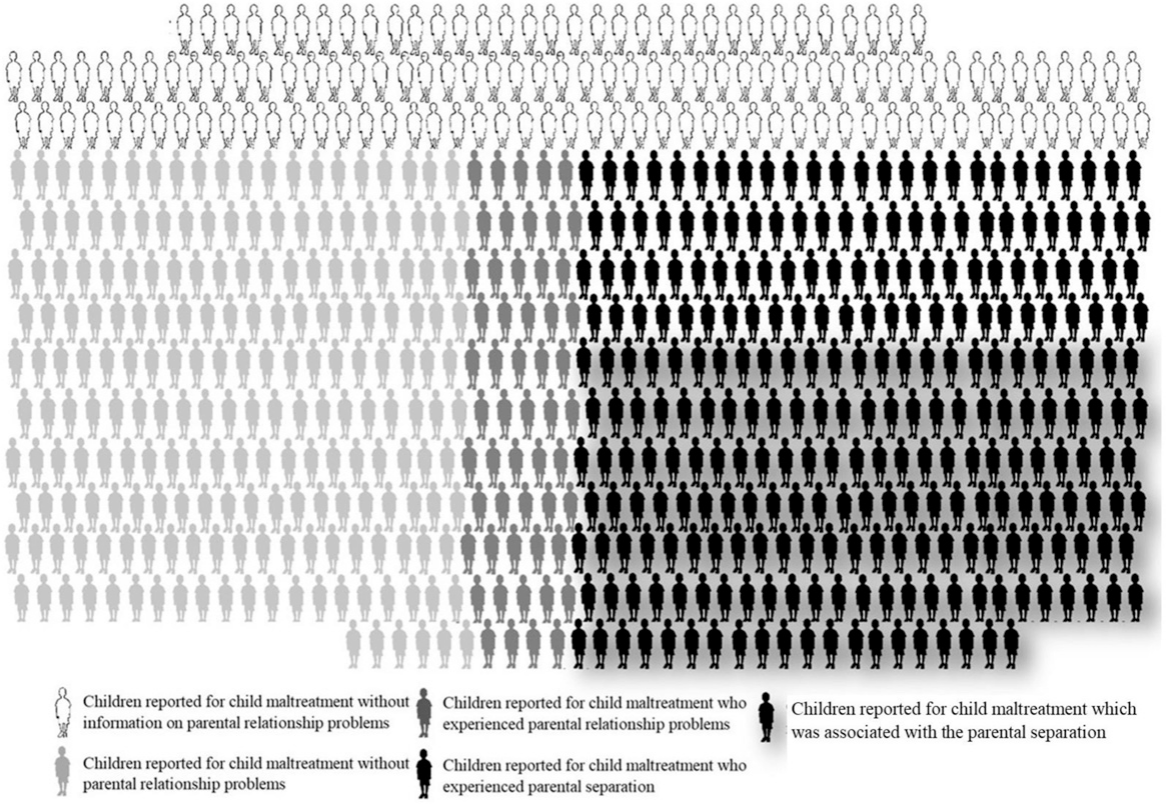
Number of children reported by sentinels for child maltreatment by reported relationship status of their parents.

Of the 663 reported cases that matched the definitions of child maltreatment, information concerning parental relationship status was available for 530 children living in 375 families. On family level, in 59% (*n =* 220) of these families a separation between parents had taken place (*n* = 190; legal divorces *n* = 122, separation between parents who were not married *n* = 68) or was about to take place (*n* = 30) according to the sentinels. On child level, 60% (*n* = 320) of the maltreated children (with available information concerning parental relationship status), had experienced or were about to experience a separation between parents (Figure 1). Of the 190 families of which the parents were separated, 64% of the separations had taken place within the past 3 years.

To test parental separation as a potential risk factor for child maltreatment, the distribution of parental separation in the general population had to be determined. Since official numbers on parental separation without parents being married are absent, it was not possible to compute a risk ratio for separation in general. Therefore, a risk ratio of legal divorce was calculated as the ratio between the proportion of maltreating families within divorced families versus the proportion of maltreating families within non-divorced families. The proportion of maltreating families within divorced families was calculated as follows: 17% of families in our sample went through a legal divorce in the years 2010–2017. The total estimate of maltreating families based on the sentinel reports was 57,224 (NPM-2017; Van Berkel et al., 2020), resulting in 9728 divorced maltreating families, versus 150,895 families in the general population who went through a divorce between 2010–2017 (proportion .064; Statistics Netherlands, 2020). The proportion of maltreating families within non-divorced families was then the remaining 47,496 maltreating families divided by the 1,612,059 families in the general population who did not divorce in this time period (proportion: .029; Statistics Netherlands, 2020). The resulting risk ratio (*RR* = 2.19; 95% *CI*: 1.37–3.49) indicates a considerable risk of child maltreatment in divorced families.

**Figure 2.**
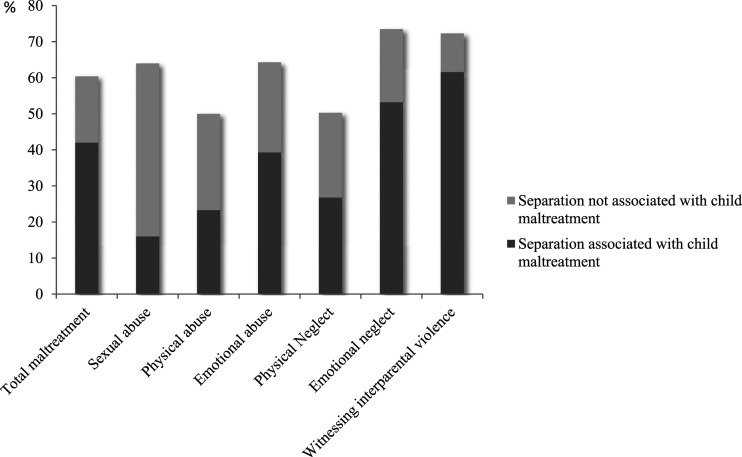
*Percentage of parental separation cases of all child maltreatment cases and per type of child maltreatment*.

The majority of the reported child maltreatment that occurred in the context of parental separation was associated with the parental separation or the impending parental separation (*n* = 220, 69%, see Figure 1). The large majority of this (82%) was only proximally associated with the (impending) separation between parents, 6% was only distally and 12% was both proximally and distally associated with the (impending) separation. Percentages of maltreatment cases that were associated with (impending) parental separations for each type of child maltreatment are presented in Figure 2. In more than half of the cases in which sentinels reported that the child had witnessed interparental violence, this form of child maltreatment was associated with parental separation (62%). In addition, 53% of the cases of emotional neglect (excl. witnessing interparental violence) were associated with parental separation, followed by 39% of the reported emotional abuse cases, 27% of physical neglect, 23% of physical abuse, and 16% of the sexual abuse cases (Figure 2).

### Child and Family Factors Associated With Separation-Associated Maltreatment

To investigate possible risk factors for parental separation-associated maltreatment, the frequencies of child and family factors in families with parental separation-associated maltreatment were compared to those in families where the reported maltreatment was not associated with parental separation using chi-square tests (Table 2). Only immigration status was associated with separation-associated maltreatment, *χ*^*2*^(2) = 6.89, *p* = .03, *V* = .21, indicating that families *without* an immigration background were overrepresented in cases of separation-associated child maltreatment. It is important to note that, given the large percentage of missing data, results concerning immigration status should be interpreted with caution.

**Table 2.** Frequencies of Child and Family Factors of Child Maltreatment Cases in Co-Occurrence With Parental Separation.

	Separation- associated child maltreatment	Child maltreatment not associated with parental separation	Missing values	Chi-Square test
Immigration background			153 (48.7%)	6.89*
No immigration background	75 (58.6%)^a^	11 (12.8%)^b^		
First-generation immigrant	17 (13.3%)^a^	8 (24.2%)^a^		
Second-generation immigrant	36 (28.1%)^a^	14 (42.4%)^a^		
Family composition			30 (13.8%)	8.75
Single-parenthood	75 (64.7%)	42 (59.2%)		
Stepfamily	11 (9.5%)	13 (18.3%)		
Co-parenting	17 (14.7%)	13 (18.3%)		
Family size (≤4 children)	8 (6.2%)	5 (6.6%)	11 (5.1%)	0.02
Child age			13 (4.1%)	1.94
0–3 years old	42 (20.0%)	21 (23.1%)		
4–11 years old	99 (47.1%)	35 (38.5%)		
12–17 years old	69 (66.3%)	35 (38.5%)		

*p < .05.

*Note.* percentages represent proportions within separation-related versus not associated with maltreatment groups. Different superscript letters denote a significant difference at the .05 level in proportions between separation-associated maltreatment and maltreatment not associated with parental separation.

### Parental Conflict Clusters

Based on the results of the two-step cluster analysis (see Method) all children with separated parents were classified into four parental conflict clusters, presented in Table 3. The division of cases in which the child maltreatment was associated with parental separation over these four clusters was as follows: (1) the *No observed conflict* group (*n* = 14, 8% of parental separation-associated maltreatment cases), (2) the *Non-physical conflict* group (*n* = 78, 33% of parental separation-associated maltreatment cases), (3) the *Verbal and physical conflict* group (*n* = 72, 36% of parental separation-associated maltreatment cases), and 4) the *Multiple conflict* group (*n* = 56, 23% of parental separation-associated maltreatment cases).

**Table 3.** Description of the Parental Conflict Clusters.

	Description	Proportion of all separated families in maltreatment sample	Proportion of parental separation- associated child maltreatment
No observed conflict	• no conflicts	99 (31%)	14 (8%)
Non-physical conflict	• verbal conflicts • legal conflicts • loyalty conflicts • parenting conflicts	81 (26%)	78 (33%)
Verbal and physical conflict	• physical conflicts • verbal conflicts	78 (25%)	72 (36%)
Multiple conflict	• physical conflicts • verbal conflicts • legal conflicts • loyalty conflicts • parenting conflicts	56 (18%)	56 (23%)

**Figure 3.**
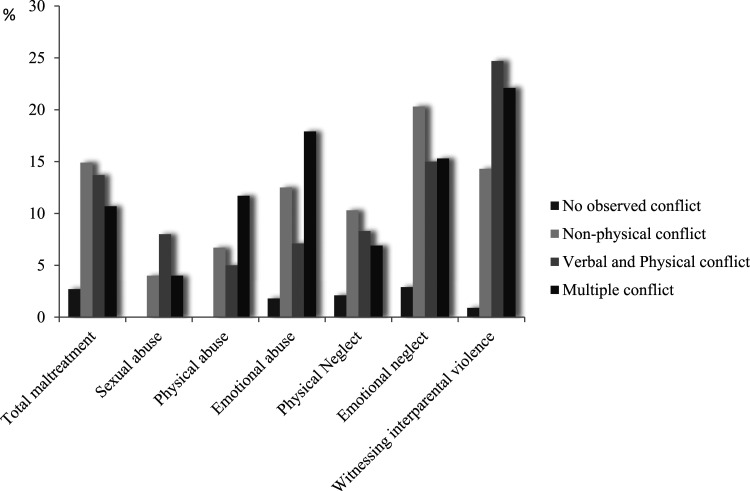
*Percentage of conflict cases of all child maltreatment cases and per type of child maltreatment (only for cases where the maltreatment was associated with the separation)*. *Note.* Prevalence estimates for the different types of maltreatment do not sum up to the total, because children may have experienced more than one type of maltreatment.

At child level, associations between the parental conflict clusters and the type of child maltreatment were computed for the cases in which the maltreatment was associated with the parental separation (Figure 3). Chi-square tests indicated that emotional neglect, *χ*^*2*^(3) = 20.83, *p* < .001, *V* = .26, and witnessing interparental violence, *χ*^*2*^(3) = 96.52, *p* < .001, *V*= .55, were associated with specific parental conflict clusters (Figure 3). Bonferroni-corrected post-hoc tests indicated that emotional neglect associated with parental separation occurred most often in the clusters *Non-physical conflict* and *Multiple Conflict,* followed by the *Verbal and physical conflict* cluster and occurred least often in the *No observed conflict cluster*. Witnessing interparental violence was, as can be expected, overrepresented in the *Verbal and physical conflict* and *Multiple conflict* clusters. Sexual abuse, *χ*^*2*^(3) = 0.76, *p* = .85, physical abuse, *χ*^*2*^(3) = 5.27, *p =* .15, emotional abuse, *χ*^*2*^(3) = .16, *p* = .15, and physical neglect, *χ*^*2*^(3) = .27, *p* = .97, were not associated with specific clusters.

**Child and family factors**. Finally, associations between parental conflict clusters and child and family factors were computed for the cases in which the maltreatment was associated with the parental separation. Chi-square tests indicated that child age, *χ*^*2*^(6) = 21.68, *p* = .001, *V* = .23, and immigration status, *χ*^*2*^(6) = 24.62, *p* < .001, *V* = .31, were associated with specific conflict clusters. Regarding child age, Bonferroni-corrected post-hoc tests showed that 0-3-year-old children were overrepresented in the *Verbal and physical conflict* cluster compared to the *Non-physical* and *Multiple conflict* clusters, whereas 12-17 year-old children were overrepresented in the *Non-physical conflict* cluster compared to the *Verbal and physical conflict* cluster. In addition, Bonferroni-corrected post-hoc tests indicated that second-generation immigrant children were overrepresented in the *No observed conflict* and the *Verbal and physical conflict* groups in comparison with the other clusters. Children without an immigration background were overrepresented in the *Non-physical conflict* cluster and the *Multiple conflict* cluster compared to the other clusters. The two family factors were not associated with specific conflict clusters: large family size, *χ*^*2*^(3) = 5.10, *p =* .17, and family composition, *χ*^*2*^(9) = 16.29, *p =* .06.

## Discussion

This study shows that the majority of children experiencing child maltreatment were also exposed to parental separation. In most of the child maltreatment cases where also parental separation occurred, the child maltreatment was associated with the parental separation. Particularly, cases of emotional neglect and emotional abuse were coded to be associated with parental separation. Moreover, emotional neglect and witnessing interparental violence occurred more often in families with separation-associated parental conflict compared to families in which interparental conflict occurred to a limited extent. Finally, there was scant evidence for associations between specific child and family characteristics and separation-associated child maltreatment or variation in the type of separation-associated interparental conflict (e.g. physical and verbal conflict, loyalty conflicts, legal conflicts, and parenting conflicts).

The results of this study suggest that parental divorce occurs twice as often in maltreating families compared to the general population. This finding is in line with evidence that parental separation is related to increased parental stress, disruptions of the parent-child relationship, and diminution of parenting quality (Amato, 2010; Lansford, 2009; Van Dijk et al., 2020), which are all factors that in turn may increase the risk on child maltreatment. In addition, this result confirms the finding of previous studies that parental separation was related to increased odds of child maltreatment (Afifi, et al., 2009; Dong et al., 2004), and strengthens the broadly supported idea that family counselors and mental health professionals should provide special attention to possible dysfunctional parenting and interparental conflict in families facing parental separation. It is also possible that families reported for child maltreatment might improve their parenting when receiving interventions aimed at diminishing conflicts concerning (co-)parenting (e.g., Mitcham-Smith & Henry, 2007). Thus, families facing parental separation as well as maltreating parents with considerable interparental conflict may improve their parenting when they receive specific interventions. Currently however evidence-based parenting interventions in families facing parental separation are lacking (Amato, 2010). More research on the effectivity of such programs is needed. It is important to note that the association between parental separation and maltreatment may also be explained by a lurking variable, composing a risk for the emergence of both parental separation and child maltreatment, for example parental psychopathology or parents’ experiences of child maltreatment. Furthermore, it is important to note that our results do not mean that the majority of parental separations would be associated with child maltreatment. In fact, in most separated families maltreatment does not occur.

This study is, to our knowledge, one of the first to cluster families based on the type of post-separation conflicts instead of differentiating between high-conflict versus normal-conflict separations (Birnbaum & Bala, 2010). Based on the type of separation-associated conflicts, we were able to distinguish four conflict clusters: a no observed parental conflict, a non-physical conflict, a verbal and physical conflict, and a multiple conflict cluster. Similar to the literature concerning the negative effects of parental separation and conflict on parenting quality (for reviews see Amato, 2010; Lansford, 2009), the results of our study indicate that interparental conflict might be a key factor relating parental separation to child maltreatment. This is reflected by the finding that in the large majority of the families in the non-physical conflict, verbal and physical conflict, and multiple conflict clusters, the reported child maltreatment was (proximally or distally) associated with parental separation (i.e., 96% of the families in the non-physical, 78% of the verbal and physical, and 100% of the families in the multiple conflict cluster; Table 3). In contrast, in most of the families in the no observed conflict cluster child maltreatment was not associated with the parental separation (i.e., only 14% of these cases were associated with parental separation). This may imply that it is not primarily the parental separation, but more so the interparental conflict associated with the separation that poses a risk for child maltreatment. However, to be able to draw this conclusion future research should compare families after parental separation without existing child maltreatment and with existing child maltreatment. The association between separation-related interparental conflict and child maltreatment adds to the literature on risks on child development of high-conflict divorces (Davies & Cummings, 2006; Fabricius & Luecken, 2007).

The finding that emotional neglect, emotional abuse, and witnessing interparental violence in particular were associated with parental separation can be explained by the literature linking interparental conflict to emotional abuse and emotional neglect (Glaser, 2011; Kelmendi et al., 2019; Van Berkel et al., 2020). High levels of interparental conflict have been associated with less parental supervision and emotional availability, and more hostile communication towards the children, which in its most extreme forms can be considered emotional abuse and neglect (Glaser, 2011; Sturge-Apple et al., 2006). This link between interparental conflict and emotional abuse and neglect is also reflected by our finding that these types of child maltreatment occurred more often in the three conflictual clusters then in the no observed conflict cluster.

When interpreting the results, it is important to keep some limitations of this study in mind. First, the data was gathered trough sentinels. Although the use of sentinels allowed us to include unreported cases of child maltreatment, this method also resulted in a considerable amount of missing data on family characteristics because some specific information on the family was not known by the participating professionals. Some groups of sentinels saw the children and their families only during occasional visits (e.g., GPs, well-baby clinics, child protection professionals of hospital) and there was variation in how systematically sentinels collected or were knowledgeable of background information on the family. This led to rather large amounts of missing data, which was to some extent associated with occupational branch. Therefore, we could not use some of the background variables in our analyses. This use of list-wise deletion may have introduced some bias given that missing background information was associated with occupational branch.

The variety in the extent to which professionals had contact with the children and their parents may indicate that access to information on interparental conflict and the extent to which child maltreatment was associated with parental separation might also differ between professionals. We tried to reduce this potential bias by asking professionals to provide detailed descriptions of the family situation and circumstances in which the child maltreatment took place and using trained coders to indicate whether the description of the sentinel could be considered child maltreatment, if there was interparental conflict, and whether the child maltreatment was associated with the parental separation. However, given the hidden nature of interparental conflict, it may very well be that interparental conflict was underreported or that a professional was not aware of the relation between parental separation and child maltreatment.

On the other hand, professionals might be biased concerning parenting problems in families with separated parents. In that case, we may have overestimated the strength of the association between maltreatment and separation. We tried to reduce this bias in two ways: (1) reported cases were coded by the same definitions that were used in other large national prevalence studies (Sedlak et al., 2010; Euser et al., 2013); (2) the relation between maltreatment and parental separation was coded by trained researchers. Finally, the difference in data-collection procedure between the CPBs and the other sentinels may have been a limitation.

In line with the described limitations, findings concerning child and family factors in relation to separation-associated child maltreatment and specific parental conflict clusters should be interpreted with caution given the large amount of missing data. Maltreated children without an immigration background more often experienced separation-associated child maltreatment compared to children with an immigration background and when they did, they were overrepresented in the *non-physical* conflict group. Second-generation immigrant children who experienced separation-associated maltreatment were overrepresented in the *no observed conflict* and the *verbal and physical conflict* groups compared to the *non-physical* and *multiple conflict* group. Thus they were overrepresented in the conflicts groups that did not include legal, loyalty, and parenting conflicts. As such, these findings may suggest that first-generation immigrant parents have less legal, loyalty, and parenting conflicts compared to parents without an immigration background. However, the difference between first-generation immigrant parents and parents without an immigration background could also be explained by professionals having less information on the specific content of conflicts between parents with an immigrant background, possibly due to language difficulties or cultural differences in the extent to which it is acceptable to communicate family issues with outsiders.

The overrepresentation of parents with young children (0–3 years old) and the underrepresentation of parents with children of 12–17 years old in the *Verbal and physical conflict* group, may be explained by increases in paternal involvement after infancy (Yeung et al., 2001). As a result, there may be a larger controversy between parents how to organize co-parenting and visiting arrangements, which may lead to more legal, parenting and loyalty conflicts. The result that none of the other family factors were related to the association between child maltreatment and parental separation or specific parental conflict clusters may indicate that the risk parental separation poses on child maltreatment is rather generic instead of specific for high-risk families. The literature on effects of parental divorce emphasizes that the *extent* of the various changes in family life as a consequence of the separation determines the deterioration of parenting quality, increases in family stress and poor psychological functioning, while less attention has been given to whether and how these effects may differ between high-versus low-risk families (Amato, 2010). However, since there is some evidence that specific family characteristics are related to separation (e.g., low income and immigration background; Alola, et al., 2020; Amato, 2010; Van Huis & Steenhof, 2003) and the amount of missing data on immigration status is considerable, the results regarding the risk of various family factors on child maltreatment in separated families should be considered carefully as they may not be replicated in a larger sample or a sample without missing data.

To conclude, parental separation, especially when there is a considerable amount of interparental conflict, often co-occurs with child maltreatment. This emphasizes that interventions for maltreating families as well as for separating parents should target interparental conflicts and focus on improving post-separation parenting and safe home environments for the children. In addition, our results may imply that a considerable number of parents in the caseload of child protection agencies may need help with resolving relationship problems or problems with an ex-partner. It is clear that interparental relationship problems may be an important characteristic of maltreatment and therefore need considerable attention in interventions aimed at reducing child maltreatment.

## Note

1 Child Protection Boards (CPBs) are an executive body of the Ministry of Security and Justice. CPBs investigate situations in which the development of a child is at risk, examine which legal advice or measures are beneficial for the child’s development in child protection and youth delinquency cases. In cases of acute of severe danger for the child and/or in cases parents refuse help, CPBs will be contacted by Safe at Home organizations.
